# A Novel Routing Scheme for Creating Opportunistic Context-Virtual Networks in IoT Scenarios †

**DOI:** 10.3390/s19081875

**Published:** 2019-04-19

**Authors:** Jaime Galán-Jiménez, Javier Berrocal, Jose Garcia-Alonso, Manuel Jesús Azabal

**Affiliations:** Department of Computer and Telematic Systems Engineering, School of Technology, University of Extremadura, Avda. de la Universidad s/n, 10003 Cáceres, Spain; jberolm@unex.es (J.B.); jgaralo@unex.es (J.G.-A.); mjesusaz@alumnos.unex.es (M.J.A.)

**Keywords:** opportunistic networks, Internet of Things, routing algorithms, contextual information

## Abstract

The massive amount of traffic required by the emerging Internet of Things (IoT) paradigm can be supported by the imminent arrival of 5G next-generation networks. However, the limited capacity of resources in IoT nodes, e.g., battery lifetime or buffer space, opens a challenge to be taken into account when proposing new routing solutions on IoT scenarios with intermittent connectivity. In this paper, we propose the concept of Opportunistic Context-Virtual Networks (OCVNs). The novelty of this approach is to create virtual groups of nodes that share interests in common for routing purposes. Therefore, only the nodes that are interested in the content of the messages that are flowing throughout the network are used as relaying nodes, providing their own resources for the sake of the communication. By leveraging the use of store-carry-and-forward mechanisms, a novel routing algorithm is proposed and evaluated over two realistic scenarios. Experimental results reveal that our solution outperforms other well-known opportunistic routing algorithms in terms of delivery probability and overhead ratio, while resource usage of relaying nodes is significantly reduced.

## 1. Introduction

During the last few years, we have seen how data traffic has dramatically increased, and this trend is expected to continue over the next few years. Some reports forecast that the global IP traffic in 2020 will nearly triple the one generated in 2015 to reach 194.4 EB per month [1,2]. As these reports indicate, the increase is mainly due to the high penetration of smartphones. While this is actually a concern, it will become a real problem as soon as the Internet of Things (IoT) devices are widely deployed. Remarkably, the prediction is that between 50 and 100 billion of these devices will be connected to the Internet in 2020 [3]. Therefore, the massive amount of data produced and exchanged by sensors, devices, and the cloud in IoT network scenarios brings to light the need to redesign both networking and data storage systems [4]. Furthermore, it is expected that the role of personal devices of mobile users in IoT networks will become more and more important in the near future [5,6]. These types of devices have a series of features that make them especially challenging for current networks [7]. First, they generate a huge amount of information that is usually spread mostly within the scope of a local area [8]. Second, a subset of them, e.g., smartphones or smartwatches, are mobile nodes that can follow different mobility patterns [5]. Finally, depending on the specific type of device, purpose, location, and situation, their connectivity can be disrupted or intermittent [9].

This scenario perfectly fits within the concept of opportunistic networking, where nodes exchange messages with other nodes they opportunistically find along their path while moving, usually by means of short-range radio technologies. Indeed, using opportunistic networks in IoT environments can result in efficient and dynamic IoT system deployments [5]. However, taking into account the limited capacity of resources in IoT nodes, e.g., battery lifetime or buffer space, several questions emerge in this context, such as: Is it possible to define an opportunistic routing algorithm able to reduce the use of nodes’ resources while also achieving a good performance in terms of delivery probability? How can one leverage relaying nodes to relay the messages efficiently to their destination without increasing the overhead ratio? The goal of this work is to shed light on these issues.

Recently, the authors of this paper have been working on the situational context concept [10]. This paradigm defines a proper way to deal with the expected increase in traffic and the interactions between IoT devices and the cloud. The idea behind it is to analyze locally the contextual information that exists at a particular time and place in order to predict, in real-time, the expected behavior of IoT devices [11]. Therefore, situational context and opportunistic IoT networking perfectly fit in order to (i) improve the coordination of IoT devices and (ii) leverage nodes with interests in common to relay messages to the destination. Thus, nodes that are not interested in the content of messages that are being forwarded will not act as relaying nodes, saving therefore resources for other purposes. Thus, techniques allowing the distribution of information depending on the needs and interests of the different nodes are required in order not to overload them with specific data in which they are not interested [12].

In this paper, we first provide the definition of Opportunistic Context-Virtual Network (OCVN) as a composition of opportunistic connections between IoT nodes with similar interests, with the aim of sharing their needs and dynamically adapting themselves to the considered context. Moreover, a novel routing scheme, namely Situational and Adaptive Context-Aware Routing (SACAR), is proposed to dynamically adapt the users’ context to the opportunistic IoT network to which they belong at a particular time, while avoiding the use of unnecessary resources. In [13], we set the base of the OCVN concept and proposed a simple routing protocol focused on sharing the interests of nodes that are within the same range. The main issue of such an implementation is that the scope of the OCVN is restricted only to the communication radio of the source node. Instead, in this paper, we propose new routing algorithms (based on [13]) in which the store-carry-and-forward technique is exploited in order to create larger OCVNs. In particular, four versions of the SACAR algorithm have been implemented considering different situations, such as: (i) source and destination nodes are in contact, i.e., there is a direct connection between them; and (ii) source and destination nodes are not in contact and some type of store-carry-and-forward techniques are required [14]. For the latter case, three situations have been analyzed. The first one is based on the fact that a message is only forwarded to those nodes that have some interests in common (in order to create the OCVN). This accomplishes that nodes with no interest in the transmitted contextual information will not compromise their (limited) resources. In the second case, only the nodes that were previously in contact with the destination node can be considered as relaying ones, therefore exploiting the historical contacts between nodes. Finally, a combination of both previous approaches is also considered. Therefore, the main contributions of the present paper can be summarized as follows:

- the definition of the OCVN concept in an IoT network scenario.
- the proposal of an opportunistic routing scheme, namely SACAR, to create OCVNs with the aim of reducing the use of resources in the set of relaying nodes.
- a performance evaluation to show the effectiveness of SACAR and to compare it with different solutions retrieved from the literature.
- a multivariate analysis to analyze the impact of the number of nodes in a scenario on different network performance metrics.

To the best of our knowledge, no prior work has proposed an opportunistic routing scheme in which the selection of relaying nodes is based on the fact that they share interests in common. Results over two representative scenarios show that our proposed SACAR-based solutions outperform other well-known opportunistic routing algorithms in terms of delivery probability and overhead ratio, while the resource usage of relaying nodes is significantly reduced.

The rest of the paper is organized as follows. Section 2 overviews previous works. Section 3 introduces the situational context paradigm, while the concept of OCVN is defined in Section 4. The SACAR algorithm is described in Section 5, while in Section 6, an estimation of the resource consumption on relaying nodes is performed. A performance evaluation based on simulations is carried out over two realistic IoT scenarios in Section 7. Finally, in Section 8, some conclusions are drawn, and future works are identified.

## 2. Related Work

Opportunistic networking is a field where huge efforts have been made by the research community during the last decade [15]. However, the emergence of IoT networks opens a way to integrate both paradigms into one promising area: opportunistic IoT. These networks are formed by a large number of fixed IoT devices, spread in a given physical area, gathering information from the environment, and generating useful data for mobile applications.

The opportunistic IoT concept was first introduced in [7]. In that work, the inherently close relationship between humans and the opportunistic connection of smart things was justified, while it was discussed how effective protocols on data dissemination can be proposed considering the impact of human behavior and mobility patterns. Since opportunistic communities are formed based on the opportunistic contact nature of humans or animals, using opportunistic networks in IoT environments can result in very efficient and dynamic IoT system deployments. As an example, the use of opportunistic IoT networks is very appropriate for emergency scenarios such as natural disasters (e.g., hurricanes, earthquakes, tsunamis, etc.), since they are infrastructure-less. Nodes can communicate with each other even with the lack of 4G/5G coverage, by using the store-carry-and-forward method, where paths between nodes are dynamically built. This aspect is very relevant, since the critical objective in emergency situations is to ensure that data generated in the disaster area are correctly delivered to the group in charge of coordinating the emergency response. In [16], an analysis of the performance of opportunistic routing protocols in emergency scenarios was presented. Specific parameters, such as the number of victims, the number of mobile nodes, or the volume of data generated were compared in order to analyze their impact on the performance of routing protocols.

Another area of application where opportunistic IoT networking can be easily deployed is wildlife tracking. In this scenario, the main objective is to monitor the activity of wild animals on a remote and geographically-large habitat, where heterogeneous IoT devices are deployed both on the ground or carried by the animals to be monitored. The challenges of real-time wildlife monitoring systems are energy efficiency, a high delivery ratio, and low latency. The authors of [17] proposed a wildlife monitoring system network architecture by using a Low Power Wide Area Network (LWPAN) with LoRa technology for collection stations and opportunistic mobile networks exploiting BLE capabilities on animals’ collars. Also in the field of passive healthcare, the opportunistic IoT paradigm can be used to create a pervasive system of intelligent devices to cooperatively gather, process, and send information on patients’ lifestyle and the context around them without requiring major changes in their behavior [18,19,20].

However, several issues must be addressed in order to provide opportunistic IoT with reliable performance, e.g., the interoperability among different standards and communication technologies, or neighbor discovery in a scenario with heterogeneous IoT devices. In [21], a mobile gateway software architecture to support IoT interoperability through a smartphone-centric application was proposed. Although the proposed architecture was validated on a real testbed without excessively using hardware resources such as CPU and memory, there were limitations related to energy consumption. The authors of [22] presented a classification and a taxonomy of neighbor discovery protocols on IoT scenarios, whose the main goal was to exploit their knowledge about mobility patterns to improve the efficiency in the discovery process. Finally, in [23], the authors proposed a routing algorithm based on trajectory prediction for identifying a mobility model by analyzing the historical mobility characteristics of the nodes and selecting the ones with the best metrics, reducing the packet loss rate, and avoiding excessive energy consumption.

In addition, due to infrequent connectivity caused by the lack of network infrastructure and random mobility models followed by devices, the routing can be increasingly complex [9]. To address this problem, the authors of [5] presented MobCCN, a protocol for accessing data generated by IoT devices in the presence of Mobile opportunistic networks. Since it is compliant with the information-centric networking principles, the idea is to populate the forwarding interest base tables of the nodes to guide the propagation of interest packets towards the destinations (i.e., the nodes that store the required data). Therefore, resource inefficient schemes are not used to propagate interest packets as in the case of traditional opportunistic routing schemes, such as epidemic routing [24].

Other research has focused on the application of machine learning technologies for improving routing solutions. For instance, in [9], a new routing protocol, called Gaussian Mixture Model Routing (GMMR), was proposed by applying Gaussian Mixture Models (GMM) and machine learning-based soft clustering mechanisms. GMMR is a method focused on flooding messages to a cluster of similar devices identified by GMM clustering. The features used to train the GMM clustering model help to identify devices with a good message forwarding character. Nevertheless, these approaches require a training phase for clustering the different nodes/devices to which the messages should be forwarded.

In [25,26], the authors implemented a fuzzy-based system for IoT device selection in opportunistic IoT networks. This algorithm uses different parameters, such as message timeout ratio, contact duration, device storage, device waiting time, and device remaining energy, in order to select the best devices to which the messages should be forwarded. This protocol allows the authors to fairly select the devices depending on their capabilities; however, it does not take into account the interests of the devices in storing and managing data.

Recently, other routing algorithms for Delay-Tolerant Networks (DTNs), such as [27], focused on an effective energy-oriented path selection and message scheduling defined for reducing the resource consumption. With the goal of reducing the overhead, Geographic-Based Spray-and-Relay (GSaR) [28] and Trajectory-Driven Opportunistic Routing (TDOR) [29] protocols are based on the historical geographic information and the trajectory of nodes for making routing decisions depending on the probabilities of encounters. Thus, the number of copies is reduced, and the delivery ratio is increased. Nevertheless, these approaches do not consider the nodes’ interests in order to not overload those nodes that are not related to the transmitted information. This perspective could be interesting for IoT networks in which nodes have limited resources.

Having introduced a review of related works on opportunistic IoT networking, the next section describes the situational context concept as a previous step to the definition of OCVNs in Section 4.

## 3. Situational Context

The aim of the IoT paradigm is to improve people’s lives by automating daily tasks. In order to improve how users interact with these devices and customize such automated tasks, the users’ context and the information gathered by IoT devices are used to adapt the system behavior to the users’ preferences in each specific situation. Therefore, the context and the gathered information have to be exchanged among the different devices in order to make that customization.

Regarding devices’ adaptation, different approaches, such as [30,31], have been defined to semi-automatically adapt the devices’ behavior to the user’s preferences. Most of these approaches follow a server-centric architectural style (i.e., the gathered contextual information is directly uploaded to a server, where it is computed to create the user’s virtual profile and shared with other devices). Later, this information is consumed by context-aware applications and smart-devices to adapt their behavior.

In the last few years, the increased computing and storage capabilities of smart-devices have allowed us to propose a new context-aware computing model (which is closer to a peer-to-peer architectural style). This model, first, uses the user’s mobile device to gather, store, and compute the contextual information in order to construct his/her virtual profile; and second, uses this virtual profile to adapt the behavior of IoT devices. This computing model is called situational context [10]. The situational context defines that a profile contains the following information:

- A *basic profile* containing the dated raw contextual information with the user’s status, the relationships with other devices, its history, and other data gathered by the device’s sensors.
- A *social profile* containing the results of inferences performed over the raw data. The information stored by the basic profile is processed by an inference engine to obtain high level data.
- The *goals* detailing the desired status of the environment can also be deduced from the basic and social profiles by using different inference rules.
- The *skills* or capabilities that an entity has, in order to make decisions and perform actions capable of modifying the environment and aimed at achieving goals.

Considering environments in which there are different entities (things and people) and each of them has a virtual profile, the situational context can be defined as the composition of the virtual profiles of all the entities involved in a particular situation. The result of composing the virtual profiles is the combined history of the entities ordered in a single timeline, the result of high level inferences performed over the combined virtual profiles, the set of goals of the entities, and their skills.

Currently, there are different programming paradigms, such as Context Oriented Programming (COP) [32], that can be used to define different behaviors of an application depending on the identification of specific contextual information. They concur that the information and/or variables triggering the adaptation are detailed within the source code of the applications. Thus, the adaptation capabilities are limited to the set of contexts defined during the system design. Instead, in the situational context, once the profiles are composed, the ways in which the entities will be coordinated to better satisfy the users’ goals are identified at run-time. Thus, the situational context provides a higher level of automation of smart things with people.

**Figure 1:**
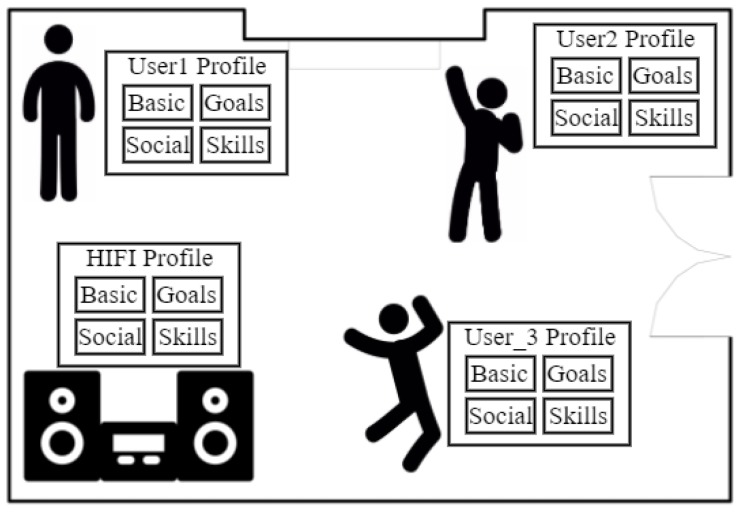
Example of the situational context.

Figure 1 shows a simple example of a living room in which there is a small party with three people and a smart HIFI system. Each person, through his/her smartphone, has a virtual profile detailing historical contextual information (e.g., the music he/she usually plays, her/his location, etc.), her/his preferences (e.g., her/his musical interests in each specific location), and her/his goals (e.g., listen to specific music styles). The smart HIFI system has its own virtual profile detailing historical contextual information (e.g., the music it has played), the goals it has (e.g., to save energy), and its skills (e.g., to play music). The situational context would be the composition of the four virtual profiles. From this composed virtual profile, the strategy to coordinate the different entities and identify what functionalities/skills should be executed are identified at run-time. Concretely, the music style and, even, the concrete songs that the HIFI system should play would be detected. The different subsets of information constituting the virtual profiles also allow us to address some problems related to the devices’ heterogeneity, since devices communicate, exchange information, and interact on the basis of two fixed concepts: goals and skills. This allows us to transmit information and coordinate the actions that must be done considering their specific capabilities. Some initial work in this direction has already been published in [33].

However, this concept has a key open issue: How are the users’ goals distributed to the entities with skills? As detailed below, the intermittent connectivity among devices, the mobility pattern of some devices, and the huge amount of information that has to be distributed make this computational model especially challenging for current IoT networks. Therefore, in this paper, we propose the use of opportunistic IoT networks and novel routing algorithms to allow users to share such information among opportunistic communities formed by IoT devices based on their movement. The next section defines a novel concept for managing opportunistic IoT networks in which situational context is applied.

## 4. Opportunistic Context-Virtual Networks

In this section, the Opportunistic Context-Virtual Network (OCVN) concept is defined. In order to describe it, a set of additional concepts related to the main components of the considered network environment must be previously defined:

**IoT device or node:** An IoT node is a general IoT device that has, at least, one wireless network interface, independent of the specific nature of the wireless technology. Two types of nodes are considered: (i) a fixed IoT node capable of gathering information from the environment (e.g., humidity sensor, presence sensor, etc.) and acting according to specific users’ needs (e.g., increase the temperature in the room, play a specific music playlist, etc.); and (ii) a mobile IoT node that has the ability to move through a geographical area and perform opportunistic connections with other IoT nodes by means of short-range communication technologies. Each node in the network is able to create its own Virtual profile (VP). In addition, a history heap is stored in each node with historical information about the number and duration of contacts between nodes with the same interests. In particular, this heap contains, for each contacted node, the next information: timestamp of the last contact, hardware address, skills’ ids, and goals’ ids. These skill ids and goal ids allow us to improve the behavior of the virtual network when it is formed by heterogeneous devices from different vendors and with different capabilities.

**Opportunistic Context-Virtual Network (OCVN)**: An OCVN is a virtual network composed of a set of IoT nodes where: (i) all of the nodes share interests (goals/skills) in common; and (ii) it is possible to send information from one node to other nodes by means of opportunistic interactions. From a general point of view, nodes of an OCVN can be either placed at the same geographical area at a given time slot or not share the same spatio-temporal features. An example of the latter would be when two nodes were connected during a period of time, shared their VPs, and after a while, one of them left the physical place where they were located (e.g., a room). Each node will store information about the VPs of the rest of the nodes in the OCVN during a certain period of time. The reason for performing this storage is to (potentially) forward these VPs to other nodes in the future, which could be interested in the goals/skills of the nodes of the former OCVN.

**Figure 2:**
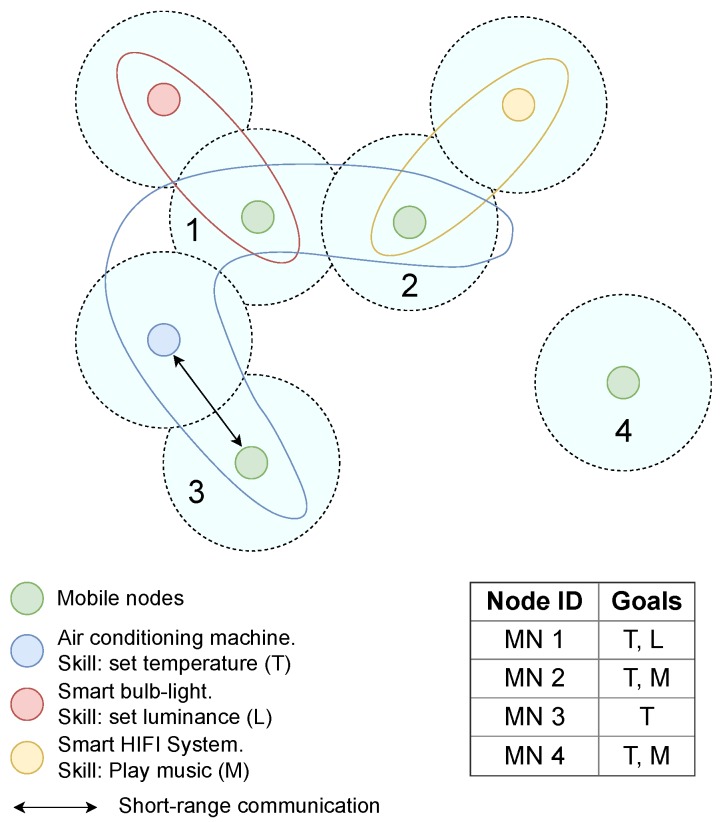
Example of three Opportunistic Context-Virtual Networks (OCVNs) in a seven-node environment.

In order to better understand the concept of OCVN, Figure 2 shows an example where seven IoT nodes coexist in the same geographical area and three different OCVNs are created. Green nodes are mobile nodes (e.g., smartphones), and their goals are described in the table shown at the right side of the figure. Regarding the set of fixed heterogeneous IoT nodes, they represent an air conditioning machine with the skill of setting the temperature, a smart light-bulb to adjust the luminance, and a smart HIFI system able to play music according to musical preferences. In this example, we assume that the coverage range, represented by dashed-line circles, is the same for each node, and a short-range communication is performed when two nodes get in contact. Colored lines represent the limits of each OCVN. It can be seen that, although Mobile Node 4 (MN4) is isolated, and hence it does not belong to any OCVN, it can still store information about the last OCVN it joined. For instance, if it is moving towards the south of the scenario, it could have belonged to the yellow OCVN in the past since one of its goals was a specific type of music. In this way, MN4 is able to store information of such OCVN during a predefined period of time to be potentially shared with other nodes in the future.

Therefore, in our OCVN-based scenarios, the use of expiration timers is mandatory to reduce both network overhead and the use of storage hardware resources at nodes. In particular, the information related to nodes that have a low probability to be met again in the near future is removed from the heap of the node upon the expiration of a timer, $\Delta_{t}$.

## 5. SACAR Algorithm Description

### 5.1. SACAR Base Algorithm

In this section, the SACAR base algorithm is explained. Let us consider a generic IoT scenario at time *t*, G=(D,t), composed of a set of $d_{i}^{x_{i}y_{i}}\in D$ heterogeneous IoT nodes, where the ith node is located at coordinates ($x_{i},y_{i}$) on a particular geographical area. Moreover, each node $d_{i}^{x_{i}y_{i}}\in D$ is a four-tuple of type $d_{i}^{x_{i}y_{i}}=\left\{BP_{i},SP_{i},G_{i},S_{i}\right\}$, where $BP_{i}$ and $SP_{i}$ are the *basic* and *social profiles* of the node, $G_{i}=\left\{g_{1}^{i},g_{2}^{i},\ldots ,g_{k}^{i}\right\}$ represents the set of *goals*, and $S_{i}=\left\{s_{1}^{i},s_{2}^{i},\ldots ,s_{p}^{i}\right\}$ is the set of *skills* the node is able to perform. Note that, although each node $d_{i}^{x_{i}y_{i}}\in D$ has a $BP_{i}$ and a $SP_{i}$, the two remaining sets are not required of every IoT device, i.e., $G_{i}=\emptyset$ or $S_{i}=\emptyset$.

The behavior of SACAR is described by Algorithms 1 and 2. In particular, Algorithm 1 describes the steps required to find neighbor nodes with similar interests, while the actions to be performed upon the reception of a message are depicted by Algorithm 2. Therefore, the former case is a proactive process, whilst the latter one is reactive. Four input parameters are required by Algorithm 1: (i) the IoT scenario D; (ii) the simulation time *T*; (iii) the frequency $\omega_{t}$ by which the messages are exchanged between nodes; and (iv) the update time interval $\delta_{t}$ to execute the functions by the nodes with skills, i.e., with $S_{i}\neq \emptyset$.

The main procedure is described as follows. Each IoT node $d_{i}^{x_{i},y_{i}}$ in the scenario, which is located at position $\left(x_{i},y_{i}\right)$, is continuously listening for incoming messages (Line 4). Every $\omega_{t}$ seconds, each node obtains the list of neighbors $N_{i}$ in G that are inside its coverage range, i.e., an interaction using short-range communication technologies can be performed (Line 6). The next step to be carried out by a node $d_{i}^{x_{i},y_{i}}$ is to send basic information about its goals $G_{i}$ and skills $S_{i}$ to each neighbor (Lines 7–9). At this point, it is not necessary to send full information for each goal and skill, i.e., pairs <id,value>, since the main purpose is to identify other reachable nodes with the same interests. The last process to be proactively performed by nodes is the execution of their associated skill functions. Clearly, these functions are only executed by those nodes with skills, i.e., with $S_{i}\neq \emptyset$. Therefore, every $\delta_{t}$ seconds, one function $f(s_{p}^{i})$ is invoked for each skill $s_{p}^{i}\in S_{i}$ (Lines 11–17).

Now that the processes carried out by nodes in a proactive way have been described, it is necessary to explain the actions required upon the reception of a message. For this purpose, Algorithm 2 is proposed. Two input parameters are now required: (i) the node $d_{i}^{x_{i},y_{i}}$ that received the message; and (ii) the message $m_{j}$ received from neighbor $d_{j}^{x_{j},y_{j}}$. The procedure now is simple. The receiving node is able to extract the ids of the goals $G_{j}$ and skills $S_{j}$, as well as the id $d_{j}$ of the node (e.g., hardware address) that created the message (Line 1). If there is a match between the interests of both nodes (i.e., between both pairs of goals and skills), the full information of matching goals/skills is now sent to the neighbor identified by $d_{j}$ by means of pairs of type <id,value> (Lines 2–4).

**Algorithm 1** SACAR pseudocode description (i): share context IDs and skills’ execution.**Require:** An IoT scenario: D, simulation time: *T*, frequency $\omega_{t}$, update time interval: $\delta_{t}$1:
t=0
▹ Current time instant2:
**do**
3:    **for all** node $d_{i}^{x_{i}y_{i}}\in D$
**do**4:        $d_{i}^{x_{i}y_{i}}.listen\left(\right)$▹ Listen if incoming messages arrive 5:        **if**
$\omega_{t}$ is triggered **then**6:            $N_{i}\leftarrow d_{i}^{x_{i}y_{i}}.getNeighbours\left(G\right)$7:            **for all** neighbor node $d_{j}^{x_{j}y_{j}}\in N_{i}$
**do**8:                $sendContextId(G_{i},S_{i},d_{j}^{x_{j}y_{j}})$9:            **end for**10:        **end if**11:        **if**
$\delta_{t}$ is triggered **then**12:            **if**
$S_{i}\neq \emptyset$
**then**13:                **for all** skill $s_{p}^{i}\in S_{i}$
**do**14:                    execute $f(s_{p}^{i})$15:                **end for**16:            **end if**17:        **end if**18:    **end for**19:
**while**
t<T


**Algorithm 2** SACAR pseudocode description (ii): reception of messages.**Require:** A node: $d_{j}^{x_{j}y_{j}}$, an incoming message from a neighbor: $m_{j}$1: $\left\{G_{j},S_{j},d_{j}\right\}\leftarrow m_{j}$▹ Extract context Id and node Id2: **if**
$\left(G_{i}\cup S_{i}\right)\cap \left(G_{j}\cup S_{j}\right)\neq \emptyset$
**then**3:    $sendFullContext(G_{i},S_{i},d_{j})$▹ Send complete context4: **end if**

### 5.2. SACAR Specific Algorithms

**Figure 3:**
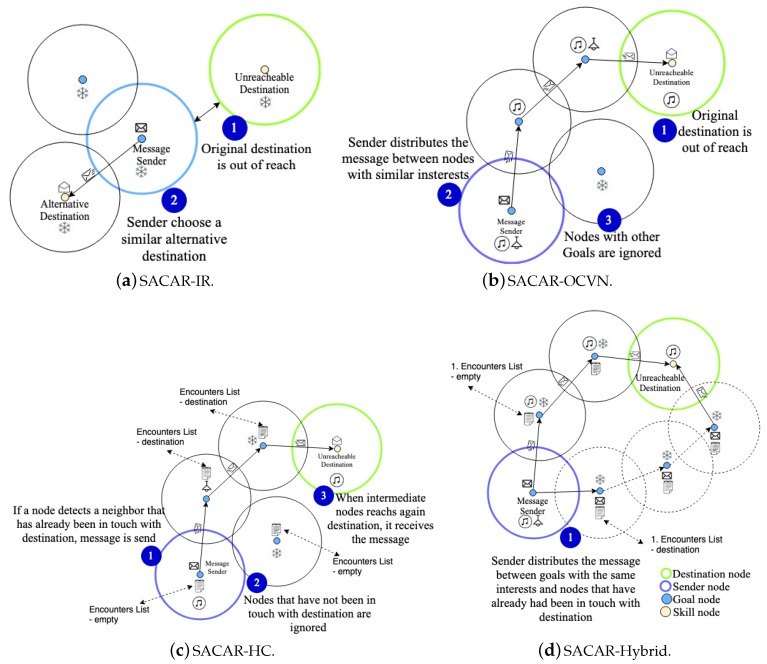
Behavior of the defined Situational and Adaptive Context-Aware Routing (SACAR) algorithms. IR, In Range; HC, Historical Contacts.

Four different versions of the SACAR-based algorithm are proposed (Figure 3) taking into account different contextual information for delivering a message (i.e., they are in contact, they share some interests, or they have previously been in the range of the destination node), namely (i) SACAR-In Range (SACAR-IR), (ii) SACAR-Opportunistic Context Virtual Networks (SACAR-OCVN), (iii) SACAR-Historical Contacts (SACAR-HC), and (iv) SACAR-Hybrid. Next, the main insights of each version are described:

- **SACAR-IR** (Figure 3a): When a message is sent by a node, before the message is created and sent (Step 1 in the figure), this algorithm first checks if the destination node is directly connected with the sender and no previous messages have been exchanged between them. If there is a connection and the destination node has some skills meeting the goals of the source node, the sender creates the message and sends it. The pseudocode of this algorithm is detailed in Algorithm 3. This algorithm has been implemented on The One Simulator [34], as is detailed in the next section. In order to implement it, this router extends the *ActiveRouter* class in order to redefine the method *createNewMessage* where the aforementioned requirements are checked. If the destination node is not located in the reachable area of the sender, SACAR-IR dynamically seeks alternative nodes that can process and adapt the environment to the sender’s preferences/goals (Step 2 in Figure 3a), checking again the defined requirements.
**Algorithm 3** SACAR-IR pseudocode.**Require:** An IoT scenario: D, simulation time: *T*, frequency $\omega_{t}$1:**do**2:    **for all** node $d_{i}^{x_{i}y_{i}}\in D$
**do**3:        $d_{i}^{x_{i}y_{i}}.listen\left(\right)$4:        **if**
$\omega_{t}$ is triggered **then**5:            **if**
$G_{i}\neq \emptyset$
**then**6:                $N_{i}\leftarrow d_{i}^{x_{i}y_{i}}.getNeighbours\left(\right)$7:                **for all** neighbor node $d_{j}^{x_{j}y_{j}}\in N_{i}$
**do**8:                    **if**
$d_{i}^{x_{i}y_{i}}.previousConnections\left(d_{j}^{x_{j}y_{j}}\right)\neq true$
**then**9:                        **if**
$d_{i}^{x_{i}y_{i}}.isConnectedTo\left(d_{j}^{x_{j}y_{j}}\right)$ and $\left(G_{i}\right)\cap \left(S_{j}\right)\neq \emptyset$
**then**10:                            **if**
$d_{j}^{x_{j}y_{j}}.getBufferOccupancy\neq full$
**then**11:                                $m_{i}\leftarrow \left\{G_{i},S_{i}\right\}$▹ The message is created12:                                $sendMessage(m_{i},d_{j}^{x_{j}y_{j}})$13:                            **end if**14:                        **end if**15:                    **end if**16:                **end for**17:            **end if**18:        **end if**19:    **end for**20:**while**t<T
- **SACAR-OCVN** (Figure 3b): This algorithm covers the situations where there is no direct connection between the sender and the destination node. Therefore, it implements the store-carry-and-forward technique to reach the destination node. When the profile has to be sent, if there is a direct connection between the sender and the receiver, the message is just created and sent. If there is no direct connection (Step 1 in the figure) with the destination node, the sender identifies the neighbor nodes sharing some common interests/goals. Then, the message is created and sent to the identified nodes (Step 2), creating the OCVN to send the message. Those nodes not sharing some of the goals are discarded. Therefore, they are not overloaded, and their resources are not wasted (Step 3 in Figure 3b), being excluded from the OCVN.
  Algorithm 4 presents the pseudocode of this routing protocol. In summary, the set of connected possible candidates to redistribute the messages and create the OCVN is checked and modified in order to fulfill one of the following requirements: the intermediate nodes share interests with the sender and, finally, one of the intermediate nodes is connected with the destination node. The implementation of this algorithm in The One Simulator mainly redefines the method *TryAllMessagesToAllConnections* to implement the aforementioned requirements.
**Algorithm 4** SACAR-OCVN pseudocode.**Require:** An IoT scenario: D, simulation time: *T*, frequency $\omega_{t}$1:**do**2:    **for all** node $d_{i}^{x_{i}y_{i}}\in D$
**do**3:        $d_{i}^{x_{i}y_{i}}.listen\left(\right)$4:        **if**
$\omega_{t}$ is triggered **then**5:            **if**
$G_{i}\neq \emptyset$
**then**6:                $N_{i}\leftarrow d_{i}^{x_{i}y_{i}}.getNeighbours\left(\right)$7:                **for all** neighbor node $d_{j}^{x_{j}y_{j}}\in N_{i}$
**do**8:                    **if**
$\left(G_{i}\right)\cap (G_{j}\cup \left(S_{j}\right)\neq \emptyset$
**then**▹ Only the nodes sharing interests are selected9:                        $m_{i}\leftarrow \left\{G_{i},S_{i}\right\}$▹ The message is created10:                        $sendMessage(m_{i},d_{j}^{x_{j}y_{j}})$11:                    **end if**12:                **end for**13:            **end if**14:        **end if**15:    **end for**16:**while**t<T
- **SACAR-HC** (Figure 3c): This algorithm also implements the store-carry-and-forward technique in order to reach nodes out of the communication range of the sender node. However, instead of sending the message to intermediate nodes that have similar interests, the message is sent to those nodes that have been previously in contact with the destination node (an OCVN is created taking into account the contextual information about the encounters among nodes). To implement this algorithm (Step 1), each node stores a list with information about the different nodes with which it has been previously in contact. Then, if the source node wants to send a message and there is no direct connection with the destination node, it searches those neighbor nodes that have previously been, or are right now, in contact with the destination node. Finally, the message is forwarded to intermediate nodes that have been/are in contact with the target node until the message reaches its destination (Step 3 in the figure).
  The pseudocode of this protocol is presented in Algorithm 5. The peculiarity of SACAR-HC is that nodes store a list with the addresses of the rest of the nodes with which they have been in contact.
- **SACAR-Hybrid** (Figure 3d): Finally, this version of the algorithm mixes SACAR-OCVN and SACAR-HC in order to be able to create OCVNs both when the nodes share some interests or when they have been in contact with the destination node (Step 1). In this case, the intermediate nodes are those that meet one of these conditions, increasing the size of the virtual network and, hence, the probabilities of the message to reach its destination.
  Given that SACAR-Hybrid combines both SACAR-OCVN and SACAR-HC, the implementation mixes these two solutions in a router implemented in The One Simulator. Concretely, the *TryAllMessagesToAllConnections* method is redefined in order to identify the intermediate nodes meeting the defined premises (at least they have a common interest or they have been in contact with the destination node). The details of this version are shown in Algorithm 6.

**Algorithm 5** SACAR-HC pseudocode.**Require:** An IoT scenario: D, simulation time: *T*, frequency $\omega_{t}$, encounters list: L1:
**do**
2:    **for all** node $d_{i}^{x_{i}y_{i}}\in D$
**do**3:        $d_{i}^{x_{i}y_{i}}.listen\left(\right)$4:        **if**
$\omega_{t}$ is triggered **then**5:            **if**
$G_{i}\neq \emptyset$
**then**6:                $N_{i}\leftarrow d_{i}^{x_{i}y_{i}}.getNeighbours\left(\right)$7:                **for all** neighbor node $d_{j}^{x_{j}y_{j}}\in N_{i}$
**do**8:                    **if then**
$d_{j}^{x_{j}y_{j}}=$ destination node OR destination node $\in d_{j}^{x_{j}y_{j}}$. L9:                    $m_{i}\leftarrow \left\{G_{i},S_{i}\right\}$▹ The message is created10:                    $sendMessage(m_{i},d_{j}^{x_{j}y_{j}})$11:                    **end if**12:                **end for**13:            **end if**14:        **end if**15:    **end for**16:
**while**
t<T


**Algorithm 6** SACAR-Hybrid pseudocode.**Require:** An IoT scenario: D, simulation time: *T*, frequency $\omega_{t}$, encounters list: L1:
**do**
2:    **for all** node $d_{i}^{x_{i}y_{i}}\in D$
**do**3:        $d_{i}^{x_{i}y_{i}}.listen\left(\right)$4:        **if**
$\omega_{t}$ is triggered **then**5:            **if**
$G_{i}\neq \emptyset$
**then**6:                $N_{i}\leftarrow d_{i}^{x_{i}y_{i}}.getNeighbours\left(\right)$7:                **for all** neighbor node $d_{j}^{x_{j}y_{j}}\in N_{i}$
**do**8:                    **if**
$d_{j}^{x_{j}y_{j}}=$ dest. node OR dest. node $\in d_{j}^{x_{j}y_{j}}.L$ OR $\left(G_{i}\right)\cap (G_{j}\cup \left(S_{j}\right)\neq \emptyset$
**then**9:▹ If the selected node is the destination or has common interests10:▹ or is in the encounter list of the intermediate node11:                        $m_{i}\leftarrow \left\{G_{i},S_{i}\right\}$▹ The message is created and sent12:                        $sendMessage(m_{i},d_{j}^{x_{j}y_{j}})$13:                    **end if**14:                **end for**15:            **end if**16:        **end if**17:    **end for**18:
**while**
t<T


### 5.3. Complexity Analysis

In this subsection, a complexity analysis for the proposed SACAR algorithms is provided. Since the main idea behind each version of the algorithm is the same, i.e., every time the parameter $\omega_{t}$ is triggered, each node in the scenario gets the list of neighbors and performs specific tasks with them, we deeply analyze the SACAR-IR approach with the focus that the same explanation can be adopted for the rest of the algorithms.

Let us then refer to Algorithm 3. Each node in the scenario $d_{i}^{x_{i}y_{i}}\in D$ (Line 2) must obtain the list of neighbors (Line 6) and perform a set of tasks for each neighbor every time the parameter $\omega_{t}$ is triggered. Since such tasks take at most O(logn), e.g., for searching in a list of previous connections (Line 8), the full process of managing the set of neighbors $N_{i}$ of a particular node $d_{i}^{x_{i}y_{i}}$ takes $O(\left|N_{i}\right|\cdot \log n)$, being $O(\left(\left|D\right|-1\right)\cdot \log n)$ in the worst-case scenario. Furthermore, if we repeat the process for each node in the network, the resulting complexity of SACAR-IR is $O(\left|D\right|\cdot \left(\left|D\right|-1\right)\cdot \log n)$ every time $\omega_{t}$ is triggered. Eventually, the complexity of SACAR-OCVN, SACAR-HC, and SACAR-Hybrid is the same as the one for SACAR-IR since the difference among them (see the pseudocode of Algorithms 4–6) is the set of functions to be executed between neighbors, taking all of them O(logn) at most.

Once SACAR algorithms have been described and their complexity has been analyzed, an estimation of resource consumption on the nodes that apply SACAR algorithms is performed in the next section.

## 6. Resource Consumption Analysis

Energy and data consumption are two important characteristics driving the success or failure of any deployment [35]. In fact, some of the authors of this paper proposed a conceptual framework to analyze during the early development stages the resource consumption of mobile devices [36]. When selecting a network architecture, it is also necessary to take into account the node’s resource consumption.

Applying the defined conceptual framework [36] to the OCVN virtual network and the SACAR algorithms, we can identify the resource consumption of each version and when each specific algorithm reduces or preserves the energy consumption. Please note that only the results of applying the conceptual framework are detailed in this section in order to improve its readability and comprehension. The resource consumption of a specific node highly depends on the following operations:

- Receiving a message (*get(size)*).
- Storing a message until it is forwarded to other nodes (*store(size)*).
- Posting or forwarding the message (*post(size)*).

The consumption of each specific operation highly depends on the size of the message. This size is the contextual information that should be exchanged among nodes, and it is subordinated to the specific domain and case study to which this concept is applied. In order to evaluate the consumption, we assumed that this size was around 768 KB. However, any change in this size would be proportional to all algorithms, so that the consumption trends would remain. The specific consumption of each operation for different sizes can be seen in the conceptual framework.

Concretely, the consumption of each node $d_{i}\in D$ also depends on the number of nodes with which it is in contact (*c*) at a specific moment (*t*) and the frequency of messages generation ω. Therefore, the consumption of each node can be calculated using the following equation (Equation (1)).

(1) $$d_{i}=\left(receive\left(size\right)+store\left(size\right)\right)\cdot \left(\omega \cdot c_{t}\right)+\left(post\left(size\right)\right)\cdot \left(\omega \cdot c_{t+1}\right)$$

**Figure 4:**
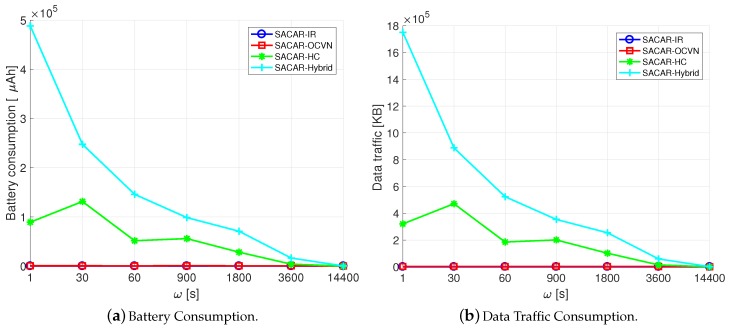
Resource consumption of SACAR algorithms.

Figure 4 shows the total battery and data traffic consumption per hour of the proposed algorithms for different frequencies of message generation. We assumed a variation of ω={1, 30, 60, 90, 1800, 3600, 14,400} s and that the total number of nodes was seven.

As can be seen, both SACAR-HC and SACAR-Hybrid are the versions that consumed the most because they spread the message to a higher number of nodes. Obviously, SACAR-IR was the approach consuming the least since it does not implement the store-carry-and-forward technique; therefore, the received messages were not forwarded. Finally, SACAR-OCVN obtained very good results regarding resource consumption since the contextual information is forwarded, but the different nodes are not overloaded. Concretely, during one hour, this algorithm only consumed 586μAH, which is 0.02% of the battery capacity of a normal mobile device.

**Figure 5:**
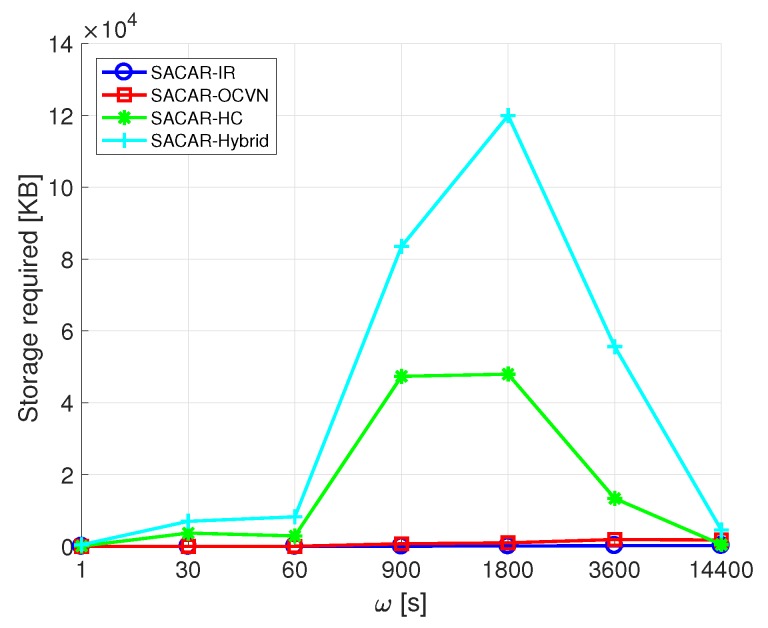
Storage required for the SACAR algorithms.

Finally, Figure 5 shows the storage required to deploy the defined algorithms. Again, SACAR-HC and SACAR-Hybrid were the most voracious algorithms regarding storage requirements. SACAR-IR was the one that presented less stringent requirements, since the messages do not have to be stored. Finally, SACAR-OCVN was the most efficient one since the received messages are stored and forwarded, but it does not require large storage capacities. Concretely, it required 1914 KB per node to store the generated messages.

## 7. Performance Evaluation

In this section, the performance achieved by the proposed SACAR algorithms is evaluated through simulations on realistic scenarios. At first, the definition of the considered scenarios and their parameters setting are provided. Next, a brief description of the algorithms that served as benchmarks is presented. Then, a performance analysis considering different network metrics, such as delivery probability, overhead ratio, average latency, and average number of hops, is carried out over each scenario. Finally, an analysis of the impact of the number of nodes with goals and the ones with skills on the network performance is also detailed.

### 7.1. Description of The Scenarios

We define a set of realistic scenarios to assess the performance of the different versions of the SACAR algorithm. We initially detail a first scenario based on a smart office. Next, we move our attention to a bigger scenario in the context of a mall.

#### 7.1.1. Smart Office

**Figure 6:**
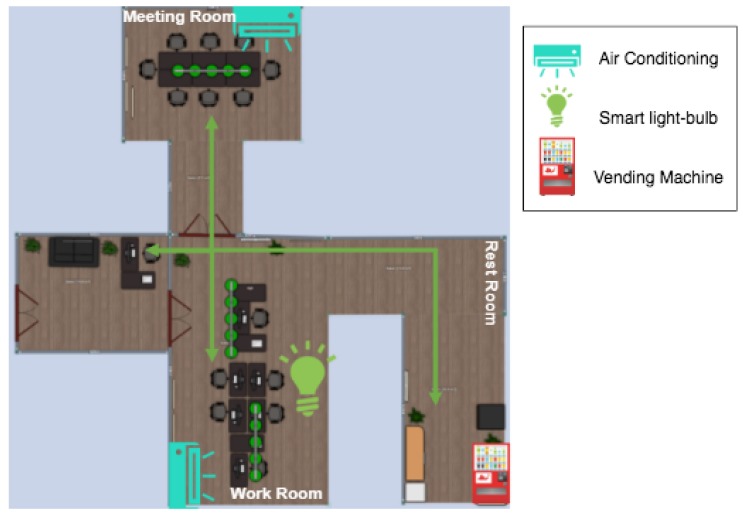
Smart office scenario.

This first scenario was composed of a set of employees with different needs and a set of smart IoT devices that were able to satisfy their requirements, i.e., they were oriented toward increasing employees’ comfort. In this scenario, different characteristics related to the social environment, i.e., the number of devices and people around, as well as the mobility pattern they usually follow during a working day, can be exploited. The concept of OCVN is, therefore, perfect for this situation. The OCVN will adapt the behavior of smart devices to the needs or preferences of employees, creating a more comfortable and productive work environment. In particular, the components composing this scenario are shown in Figure 6 and described next:

- Two air conditioning machines, one in the work room and another one in the meeting room. Both devices have the skill to modify the ambient temperature. This is done by getting the desired temperature of the employees in each room and assessing the average desired temperature. Note that, although this algorithm could be much more complex (e.g., by setting different weights to employees according to their role in the company), we kept it simple to focus on analyzing the probability of messages’ delivery with the assessed information (the analysis of more complex functions to be performed by the nodes with skills is left for future work).
- Smart light-bulbs, located in the work room, which are able to modify light intensity depending on the employees working in the room.
- A vending machine, situated in the break room, with the skill to serve drinks depending on people’s preferences.
- Seven employees with different roles and preferences regarding lighting and temperature in their workplace, as well as preferred drinks.

Table 1 summarizes the setting of the parameters in this scenario. A total of N=11 nodes were considered to be located inside an office of A=150 m${}^{2}$, $N_{s}=4$ of them being static nodes with skills, and the rest of the $N_{g}=7$ nodes are mobile ones (i.e., employees). The set of skills and the set of goals were the same, i.e., S=G={Temperature,Illuminance,ProductType}. With this setting, it is assumed that the number of mobile nodes with goals (e.g., smartphones or tablets) moving along the scenario was larger than the number of static IoT nodes able to perform actions associated with particular skills.

Regarding the number of skills per stationary node, we considered that a device was only able to manage one skill ($s_{n}=1$), i.e., it was created with a specific and unique purpose (e.g., for the case of the air conditioner, it is designed to set a particular temperature). On the contrary, a mobile node is able to consider different goals. In this scenario, the number of goals per mobile node was set to $g_{n}=3$. The possibility of considering more than one skill per device is left for future work.

Concerning the movement of mobile nodes (employees), Figure 6 also shows a draft of the mobility patterns set, *P*, considered in the scenario. As can be seen, there are two different movement flows (green lines), a first one between the meeting room and the work room and another one between the work room and the rest room (the investigation of the impact of considering additional mobility patterns is left for future work). These two mobility patterns were selected because they are the usual ones in an office. Two or three times per day, every employee goes to the meeting room to discuss specific projects, and every one or two hours, they go to the break room to have a break. Moreover, we consider that one of the employees is the secretary, with a special role able to follow different mobility patterns towards the different rooms; usually, the secretary does not go to the meeting room, but goes more frequently to the work and break rooms.

For the evaluation, the simulation duration was set to *T* = 28,000 s (the duration of an average working day), and a parameter, ω, was defined to set the frequency at which messages were generated during the simulation. As an example, if ω=60 s, messages were created and sent to their destination every minute. Table 1 shows the different values of this parameter used in order to perform independent executions and compare the obtained results as a function of it.

#### 7.1.2. Mall

**Figure 7:**
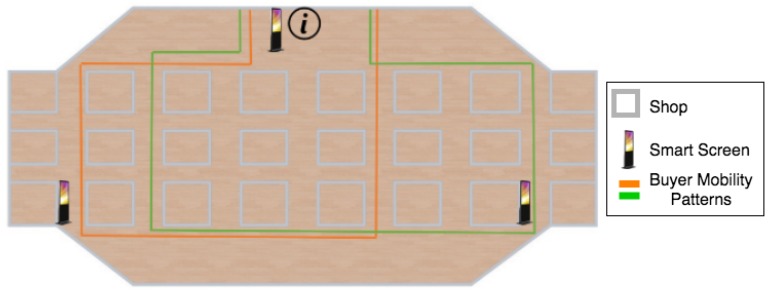
Mall scenario.

In the following, we move our attention to the definition of a bigger scenario based on a mall (see Table 2 for the specification). In this second scenario, personalized information is shown to the buyers depending on their preferences, needs, previous purchases, etc. That information is shown by different screens installed at the mall’s information point and throughout the shopping center. The exchange of contextual information between people and screens allows the shops to show personalized offers to the buyers and also the mall manager to get information such as the behavior of the customers, their profiles, and preferences. This case study aims at improving the buyers’ satisfaction by showing them a set of personalized offers, while at the same time, the visibility of those offers is also increased. By exploiting this scenario, our aim is to evaluate the behavior of the SACAR algorithms in environments with a large number of users who do not follow a clear movement pattern. In particular, the different components of this scenario are shown in Figure 7 and described next:

- Screens located throughout the mall with the skill of showing buyers different information depending on their needs, such as specific shops, products, and offers in which they may be interested.
- Buyers who have virtual profiles with different goals depending on their needs, preferences, past purchases, etc.

Figure 7 shows a draft of the simulated environment and some example mobility patterns. As can be seen, two mobility flows have been considered (green and orange lines). In the figure, only two routes have been drawn in order to increase the readability of the picture. Nevertheless, for the final simulated scenario, eight different routes were evaluated. These mobility patterns have been assigned to the buyers depending on their preferences and goals, since usually people with the same preferences visit similar types of shops. Similarly, several screens have been placed at different locations inside the mall. Nevertheless, for this scenario, we also evaluated the impact of increasing or decreasing the number of nodes with skills over the delivery probability.

The main objective of using this scenario is to evaluate the performance of the proposed solutions in an environment with a high number of nodes that can move following different mobility patterns. Moreover, we can also analyze the impact of an increase in the number of buyers (nodes with goals) on the algorithms’ performance, e.g., simulating that the mall is crowded. Moreover, a trade-off analysis on the minimum number of screens (nodes with skills) that are required to reach most of the buyers can also be performed.

### 7.2. Benchmark Algorithms

To better compare the behavior and performance of the proposed algorithms, we also used several well-known opportunistic routing algorithms found in the literature. Next, a brief description of each of them is provided:

- **Direct Delivery Routing (DDR)** [37]. This algorithm sends a message only when there is a direct communication between nodes. No messages are relayed or copied, and a direct path between source and destination must exist.
- **Epidemic Routing (ER)** [24]. In this routing algorithm, a copy of the message is sent to each neighbor. It is assumed that each node has unlimited storage space (i.e., buffer) and unlimited bandwidth.
- **MaxProp Routing (MPR)** [38]. This algorithm computes the shortest path to each destination on the basis of contact history and the number of hops. Copies are deleted when a message reaches its destination.
- **ProPHET Routing (PR)** [39]. The aim of this algorithm is to improve the routing performance by adopting the probabilistic scheme that reflects the contact observation of the nodes.
- **Spray and Wait Routing (SWR)** [40]. In this algorithm, the source node creates *N* copies of the message to spread it to relaying nodes. After that, the latter ones perform direct message transmission such in the case of DDR.
- **Geographic-Based Spray-and-Relay (GSaR)** [28]. The idea behind this algorithm is to exploit historical geographic information and the trajectory of nodes for making routing decisions depending on the probabilities of encounters.

### 7.3. Experimental Results

We have implemented SACAR-IR, SACAR-OCVN, SACAR-HC, and SACAR-Hybrid in The One Simulator [34]. The benchmark algorithms except GSaR are already included in the simulator; therefore, we decided to also implement the GSaR algorithm in order to compare the proposed routing solutions with another approach following a similar philosophy. Each algorithm was run on a laptop with 2.3 GHz 2-core Intel i5, 16 GB of RAM, and Intel Iris Plus Graphics 640.

#### 7.3.1. Evaluation of the Smart Office Scenario

The first analysis we propose is to compare the outcomes of several network metrics when applying the proposed algorithms to the smart office scenario described in Section 7.1.1. As introduced in Section 7.1, we assumed a variation of ω = {1, 30, 60, 90, 1800, 3600, 14,400} s to analyze the impact of the frequency of message generation on the network performance. Therefore, a total of |RA|*|ω| simulations have been run varying the components of pairs of type E={RA,ω}; where RA={SACAR−IR,SACAR−OCVN,SACAR−HC,SACAR−Hybrid,DDR,ER,MPR,PR,SWR,GSaR} is the set of different routing algorithms. Moreover, four network metrics were examined in each simulation: (i) average delivery probability; (ii) overhead ratio; (iii) average latency; and (iv) average number of hops to reach the destination. In the following, the definition of each metric is provided.

Let us define the number of messages that are created by node *i* and sent towards node *j* as $c_{i,j}$. Moreover, the number of delivered messages to node *j* starting from node *i* is given by $d_{i,j}$. The average delivery probability $d_{\mathrm{prob}}$ in the network is given by Equation (2):

(2) $$d_{\mathrm{prob}}=\frac{\sum_{i\in D}\frac{d_{i,j}}{c_{i,j}}}{\left|D\right|},\forall i,j\in D$$

In order to determine the performance of a routing algorithm in terms of number of relayed messages, i.e., the number of times each message is received by an intermediate node and relayed to another node in its path towards the destination, the overhead ratio θ defined by Equation (3) is considered:

(3) $$\theta =\frac{\sum_{i\in D}\frac{r_{i,j}-d_{i,j}}{d_{i,j}}}{\left|D\right|},\forall i,j\in D$$

where $r_{i,j}$ represents the number of relayed messages during the process of sending a message from node *i* to node *j*. Finally, the average latency, τ, and the average number of hops, γ, were also considered in the performance analysis to evaluate the effectiveness of each algorithm in terms of response time.

**Figure 8:**
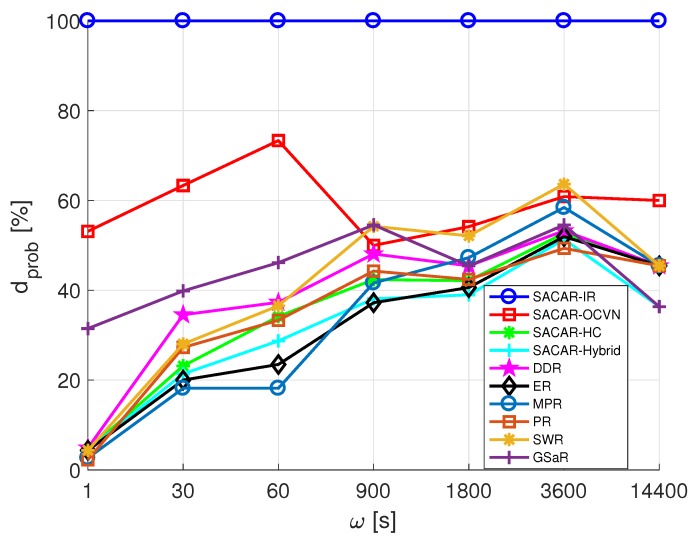
Average delivery probability ($d_{\mathrm{prob}}$) vs. the frequency of messages generation (ω): smart office scenario. DDR, Direct Delivery Routing; ER, Epidemic Routing; MPR, MaxProp Routing; PR, ProPHET Routing; SWR, Spray and Wait Routing; GSaR, Geographic-Based Spray-and-Relay.

Once the different network metrics have been defined, we focus on analyzing them in the case of the smart office scenario. As previously introduced, the delivery probability $d_{\mathrm{prob}}$ evaluates the likelihood of a message to reach its destination. Since all the nodes in the scenario are able to send messages toward the rest of nodes, Figure 8 reports the values obtained for $d_{\mathrm{prob}}$ as a function of ω.

By inspecting Figure 8, several considerations emerge. At first, it can be seen that SACAR-IR presents a 100% delivery probability regardless of the frequency of message generation. The explanation of this situation is that the logic of this algorithm is different in nature compared to the rest of the evaluated algorithms. In SACAR-IR, a source node will only send a message to a destination node that (i) is located within its action range and (ii) has the same interests (goals/skills matching). Since this solution [13] highly restricts the set of potential destinations, each created message will definitely reach its destination, with a resulting average delivery probability of $d_{\mathrm{prob}}=100\%$.

Regarding both the rest of SACAR versions and the benchmark solutions, it is necessary to remark that all of them exploit the store-carry-and-forward technique on packet forwarding. In these cases, source and destination nodes are not required to be within the same range, allowing intermediate nodes to receive a packet, temporarily store it, and forward it to a node that will (potentially) be met along the path. Looking again at Figure 8, we can notice that SACAR-OCVN is the store-carry-and-forward algorithm that presents the best results in terms of delivery probability, reaching a peak value of 73.33% for ω=60 s. The exploitation of the OCVN concept in the forwarding actions of a node makes this solution particularly appealing for scenarios such as a smart office, with a limited size and a big ratio of individuals sharing common interests. Interestingly, the rest of the algorithms revealed a similar trend: when ω was increased, i.e., the time period between rounds of messages was longer, $d_{\mathrm{prob}}$ tended to increase. In particular, for values of ω≤60 (i.e., when messages were generated very frequently), GSaR presented the best outcomes among this last set of algorithms. This algorithm presented a linear increase of $d_{\mathrm{prob}}$ for values of ω≤900. Concretely, for ω=900, it provided better results than SCAR-OCVN. In turn, SWR behaved better for values of ω≥900 s, slightly overcoming our SACAR-OCVN solution for ω=900 s and ω=3600 s. Finally, while SACAR-HC remained at the average of the benchmark algorithms, SACAR-Hybrid suffered for large values of ω.

**Figure 9:**
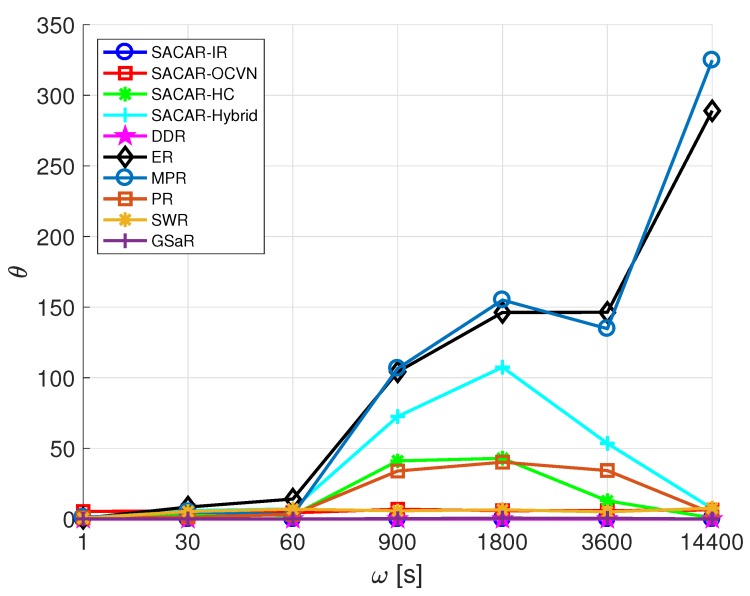
Overhead ratio (θ) vs. frequency of message generation (ω): smart office scenario.

The second metric we aimed to analyze is the overhead ratio, θ, which is reported in Figure 9 as a function of ω. From the figure, we can extract that, apart from the solutions that do not consider the possibility of relaying messages (SACAR-IR and DDR) where no overhead was experienced (θ=0), SACAR-OCVN, SWR, and GSaR presented a similar flat trend with low values of θ independently of the value of ω. This means that a very small gap between the number of relayed messages with respect to the number of delivered ones was obtained. Therefore, nodes’ resources such as buffer occupation and energy consumption due to forwarding actions were not highly impacted because of the use of these algorithms. Instead, a clear increase in the value of θ with respect to ω was experienced when using MPR and ER. The spreading logic behind these solutions, where several copies of the message are created under different circumstances, severely impacted the network performance in terms of overhead ratio.

**Figure 10:**
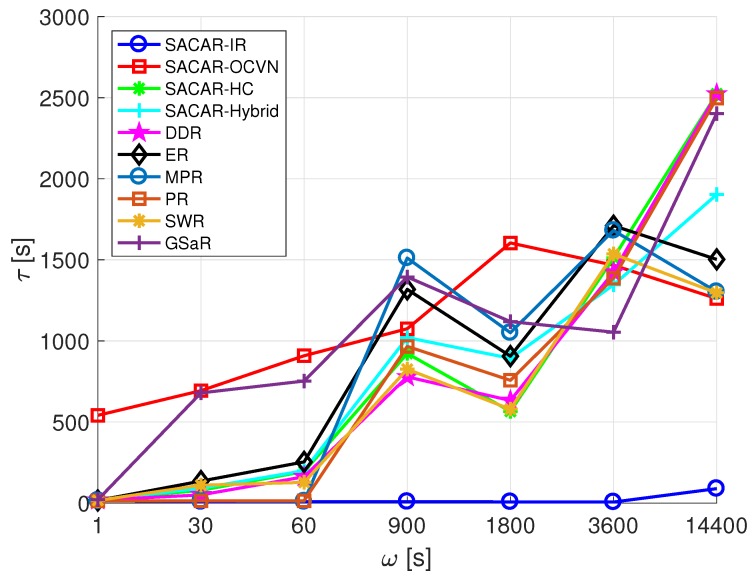
Average latency (τ) vs. frequency of message generation (ω): smart office scenario.

In the following, we evaluate the average latency, γ, experienced by a message on its way from the source to the destination node. By inspecting Figure 10, it can be seen that the average latency tended to increase with ω. Clearly, SACAR-IR presented the best results in terms of average latency, since the source-destination pair was constrained to be physically placed within the same range. If we focus on the store-carry-and-forward-based algorithm set, we can see that both SACAR-OCVN and GSaR suffered for small values of ω compared to the rest of the algorithms. The explanation of this situation for SACAR-OCVN is that a node will only forward the message to another node with the same interests (goal-skill matching). Similarly, for GSaR, a message was only forwarded if the nodes followed a specific trajectory. Therefore, although there may be several possibilities for forwarding the message when two nodes meet each other, a further constraint in the checking must be also satisfied. Remarkably, although SACAR-OCVN produced interesting results for large values of ω, SWR was the algorithm that in general best fit the average latency among the ones that allowed the nodes to have a buffer to temporarily store messages.

**Figure 11:**
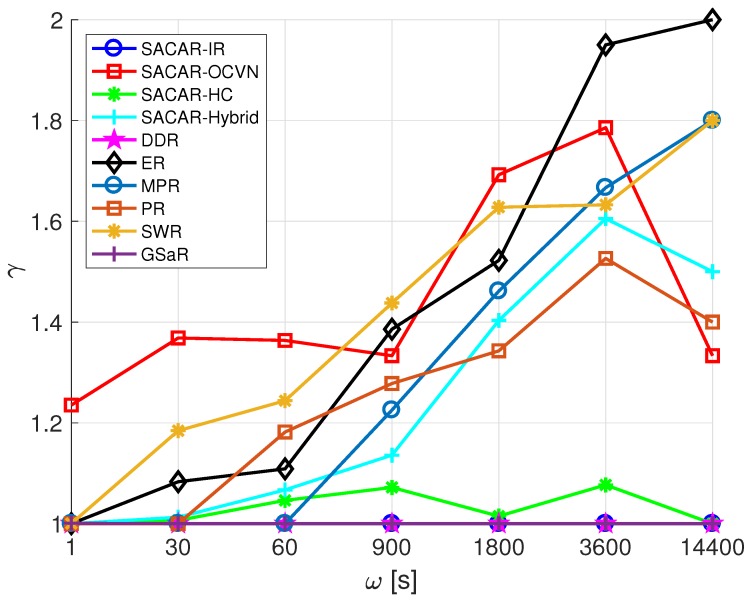
Average number of hops (γ) vs. frequency of message generation (ω): smart office scenario.

Finally, we move our attention to analyze the number of hops a message needs to take from the source node towards the destination node. Figure 11 reports the average number of hops as a function of ω. Once more, the generic trend was that the number of hops increased with ω, except for SACAR-IR, DDR (since the logic behind them is to create and send a message only to destinations that are one hop away), and SACAR-HC, for which the average number of hops was stabilized around one for each value of ω. For GSaR, the number of hops was also stabilized around one because it is focused on different types of scenarios. Remarkably, and differently from the previous analysis in which the average latency was studied, SACAR-OCVN and SWR presented comparable results in terms of the number of hops.

As a summary, after the evaluation of the proposed algorithms and their comparison with the benchmark ones in a small scenario such as a smart office, we can state the next remarks: (i) if most of the nodes are within the same range, the best solution is to use SACAR-IR; (ii) if (i) is not the case, SACAR-OCVN presents the best outcomes among the set of store-carry-and-forward routing algorithms on delivery probability and overhead ratio, although an increase in the average latency and in the number of hops is experienced; (iii) if a balanced trade-off between delivery success and latency is required, then the best option according to the obtained results is to use SACAR-HC.

Having evaluated a small scenario, in the next section, we analyze the outcomes of the proposed solutions over a bigger one, both in terms of area to cover and in the number of nodes.

#### 7.3.2. Evaluation of the Mall Scenario

A mall scenario is simulated by selecting a subset of nodes as devices with skills (screens), and the rest of them are people that go to the mall with the aim of buying items (i.e., their goal is to purchase). As in Section 7.3.1, our first objective is to analyze the performance of each algorithm in this scenario. In addition, since the routing protocols proposed in this paper take into account the similarity between the goals/skills of the encountered nodes in order to forward a message (creating the OCVN), we also evaluate the impact of increasing the number of nodes with goals and the number of nodes with skills on the network performance.

**Figure 12:**
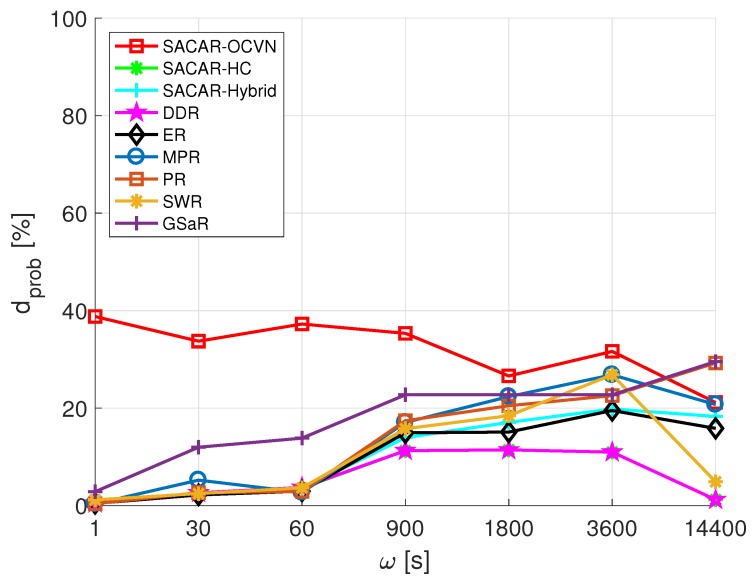
Average delivery probability ($d_{\mathrm{prob}}$) vs. frequency of message generation (ω): mall.

The first network metric we aim to analyze is the average delivery probability. The resulting values of $d_{\mathrm{prob}}$ as a function of ω are reported in Figure 12. Remarkably, SACAR-OCVN again outperformed the rest of the algorithms, especially for low values of ω. In fact, the best gains were obtained when messages were continuously generated and the network traffic was high (40% of gains when ω=1 s). Although SACAR-OCVN $d_{\mathrm{prob}}$ slightly decreased with ω, it was almost stable and always above 20%. On the contrary, the rest of the algorithms presented an increasing trend as a function of ω, but their outcomes were worse than the ones obtained when applying the concept of OCVN. Among them, GSaR must also be highlighted because it provided better results for ω≤1800. In fact, for ω = 14,400, it presented even better results than SACAR-OCVN.

In a big scenario such as a mall, it is necessary to evaluate the time a message is stored in the buffer of a node. Since there are many nodes in the network and the traffic can be significantly high, an efficient usage of nodes’ storage capabilities to perform store-carry-and-forward actions must be performed. Therefore, a new metric is analyzed in this scenario, δ, which is the average time a message is stored in a buffer (since the obtained results for the rest of metrics considered in Section 7.3.1 presented similar conclusions in the mall scenario, we avoid to including them in this analysis, and we only focus on $d_{\mathrm{prob}}$ and δ).

**Figure 13:**
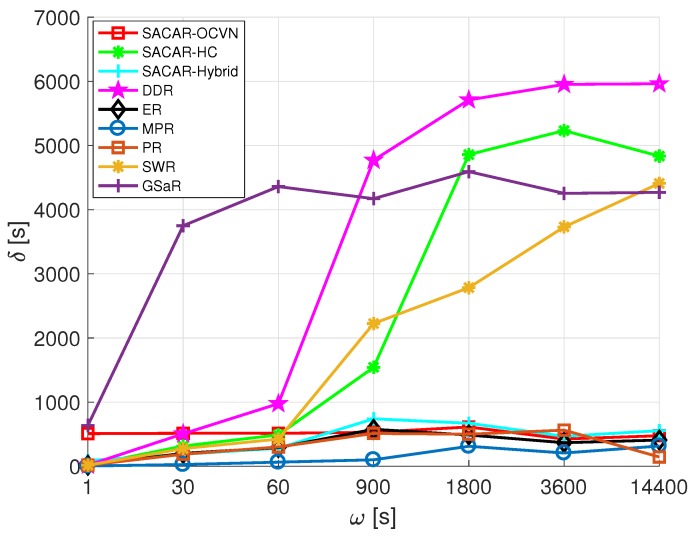
Average buffer time (δ) vs. frequency of messages generation (ω): mall.

Figure 13 reports the values of δ as a function of ω. From the results, we can highlight that a division of the algorithms into two groups according to their behavior respecting δ can be done. At first, there were four solutions (DDR, SACAR-HC, GSaR, and SWR) for which the average time a message spent inside a relaying node increased with ω, reaching prohibitive results when the time period between consecutive rounds of message generation was high. On the contrary, there was no impact on δ when ω was varied for the rest of algorithms. If we focus on our best solution in terms of delivery probability, i.e., SACAR-OCVN (see Figure 12), we can state that, although the logic of the algorithm restricts the number of nodes that can be used for relaying a message (there must be a goals/skills matching), and therefore messages must spend longer times in buffers, δ results reported in Figure 13 indicate that these times are comparable with solutions where such a constraint is not taken into account and where messages can be relayed by any node found along the path.

In the last analysis, our aim is to evaluate what is the impact of increasing the number of nodes with goals and the number of nodes with skills on the different considered metrics when our SACAR-OCVN solution is applied. To do that, a multivariate analysis has been performed considering the number of nodes having goals $N_{g}$, as well as the number of nodes with skills $N_{s}$ in the scenario as independent variables, while the considered dependent variables were $d_{\mathrm{prob}}$, θ, τ, γ, and δ. Thus, the idea is to know the impact of each independent variable on the selected dependent variables. As a summary, the set of variables used in the statistical analysis are defined and categorized in Table 3.

Regarding the setting of the simulations to obtain the results to be statistically analyzed, we considered the mall scenario with a value of ω=3600 s, i.e., the time period between two consecutive rounds of messages creation was one hour. This interval was selected due to the fact that it allows the nodes to move along the scenario and exploit their store-carry-and-forward capabilities while no other aspects can interfere with the algorithm’s functioning, e.g., an increase in the amount of traffic flowing through the network. Several tests were performed by varying the number of nodes and the initial position of each of them in the scenario. In fact, a total of 25 independent runs were carried out for each test to retrieve statistically-significant outcomes.

Table 4 shows the outcomes after performing the Ordinary Least Squares (OLS) regression considering the number of nodes with goals as independent variable, achieving an average value of $R^{2}=0.876$. First, all the variables considered in the analysis were statistically significant (p<0.05) except $d_{\mathrm{prob}}$, for which no conclusions can be drawn (p=0.265). Looking at unstandardized coefficients (B column), it is clear that there existed a directly proportional relationship between the number of nodes with goals, $N_{g}$, and the overhead ratio, θ, experienced in the network. In particular, an increase in one unit in the number of nodes with goals in the scenario was associated in a statistically-significant way with an increase in 1.719 in the overhead ratio. In the same way, an increase of 7.168 s on the average latency, τ, was paid as a penalty if a new node became part of the network. The number of hops, γ, in turn, was not highly affected by the considered independent variable, with a slight increase per added node. However, it is interesting to highlight that the only variable with negative (but also statistically significant) coefficients was the average buffer time, δ, with B=−1.363. This means that buffer time was reduced with the increase in the number of nodes with goals, following a decreasing progression of 1.363 s per new node. This situation can be explained as follows. Since there are more nodes in the network to share common interests with, the probability for a node to store a message temporarily that is intended for a different destination decreases. Therefore, the average buffer time in the network also decreases.

Similar results emerge by inspecting Table 5, when the number of nodes with skills, $N_{s}$, is taken as the independent reference variable. Again, an increase in the overhead and in the average latency was obtained as the result of adding a node with skills to the network; whilst a reduction of more than four points in the average buffer time was achieved. Similarly to Table 4, the impact of increasing the number of nodes over the number of hops a message must traverse remained negligible (B=−0.047). However, the main difference with the previous analysis was that the $d_{\mathrm{prob}}$ variable was statistically significant. Therefore, we can extract from the results that the impact of adding a node with skills to the network is associated with an improvement of 1.521% in the delivery probability.

To sum up the OLS regression outcomes, the higher the number of nodes in the network, the higher the overhead ratio and the average latency imposed by the application of SACAR-OCVN. However, a remarkable point is that nodes’ resources, such as their buffer to store messages temporarily, were efficiently used by our proposed solution. Finally, the delivery probability was increased in the case of adding nodes with skills to the network.

## 8. Conclusions and Future Work

The increase in the number of Internet-connected smart devices and the deployment of the IoT paradigm in the forthcoming 5G era has raised the need for these devices to behave and coordinate themselves depending on the interests and needs of their users. Therefore, the network has to provide support to this exchange of information in environments where devices have some mobility and the connections among them is intermittent, depending on their concrete location and the used communication interface.

This paper proposes the creation of Opportunist Context-Virtual Networks (OCVNs) based on the devices’ contextual interests and their capabilities. In OCVNs, IoT devices with similar interests can share their virtual profiles and adapt themselves to the considered context. In particular, four novel routing algorithms, namely SACAR-IR, SACAR-OCVN, SACAR-HC, and SACAR-Hybrid, were proposed to exchange the users’ context and interests to the OCVN they belong to at a particular time dynamically. These algorithms allowed us to improve the exchange of contextual information in IoT environments with mobile nodes, without overloading those IoT devices not interested in those data; improving, thus, the behavior of the whole system.

The proposed algorithms have been evaluated over two representative scenarios based on an office and a mall with IoT smart devices. These scenarios were chosen because they represent common IoT deployments in which end users are involved, first at a personal level in small areas (such as their home or office) and, second, in medium-sized areas (such as malls, concerts, etc.). Simulation results show that SACAR algorithms outperformed other well-known opportunistic routing algorithms in terms of delivery probability and overhead ratio, while the resource usage of relaying nodes was significantly reduced. Moreover, the multivariate analysis (OLS regression) performed to know the impact of the number of nodes over several performance metrics also reinforced the conclusion that nodes resources were efficiently used by our proposed solution.

As future work, we are currently working on applying machine learning techniques in order to use more complex contextual information to perfectly identify those nodes that should belong to a particular OCVN. In addition, since IoT networks are also used in large scenarios with a high number of nodes and large geographical coverage, we are also working on evaluating these algorithms in more complex environments such as agriculture and emergency scenarios in which a higher number of nodes is involved with a larger variability of interests. Finally, cyber-security threats are specially challenging in opportunistic and delay-tolerant networks due to their self-organizing nature and uncertainty in the nodes composing them. These issues are even more challenging in environments in which contextual and sensitive information is shared, such as presented in this paper. Therefore, we are also analyzing some of the state-of-the-art approaches, such as [41,42,43], in order to incorporate security aspects into the presented algorithms.

## Figures and Tables

**Table 1:** Parameter setting for the smart office scenario.

Parameter	Value
*A*	150 (m${}^{2}$)
*N*	11
$N_{s}$	7
$N_{g}$	4
*S*	{Temperature, Illuminance, ProductType}
*G*	{Temperature, Illuminance, ProductType}
$s_{n}$	1
$g_{n}$	3
*P*	{StationaryMovement for $N_{s}$, MapRouteMovement for $N_{g}$
*P*	with working time periods in the range of t=[1800,7200] s}
*T*	28,000 (s)
ω	{1, 30, 60, 90, 1800, 3600, 14,400} (s)

**Table 2:** Parameter setting for the mall scenario.

Parameter	Value
*A*	225,000 (m${}^{2}$)
*N*	41
$N_{s}$	3
$N_{g}$	38
*S*	{Purchases}
*G*	{Purchases}
$s_{n}$	1
$g_{n}$	1
*P*	{StationaryMovement for $N_{s}$, MapRouteMovement for $N_{g}$
*P*	with stationary time periods in the range of t=[300,500] s}
*T*	28000 (s)
ω	{1, 30, 60, 90, 1800, 3600, 14,400} (s)

**Table 3:** Criteria and variables used in the multivariate analysis.

Variable	Description	Type
$N_{g}$	Number of nodes having goals	Independent/continuous
$N_{s}$	Number of nodes having skills	Independent/continuous
$d_{\mathrm{prob}}$	Average delivery probability	Dependent/continuous
θ	Overhead ratio	Dependent/continuous
τ	Average latency	Dependent/continuous
γ	Average number of hops	Dependent/continuous
δ	Average buffer time	Dependent/continuous

**Table 4:** OLS regression coefficients with $N_{g}$ as the independent variable.

Variable	*B*	Std. Error	Beta	*t*	Sig.
$d_{\mathrm{prob}}$	−0.025	0.023	−0.113	−1.122	0.265
θ	1.719	0.141	0.777	12.228	0.000
τ	7.168	1.340	0.475	5.348	0.000
γ	0.023	0.001	0.898	20.164	0.000
δ	−1.363	0.207	−0.553	−6.568	0.000

**Table 5:** OLS regression coefficients with $N_{s}$ as the independent variable.

Variable	*B*	Std. Error	Beta	*t*	Sig.
$d_{\mathrm{prob}}$	1.521	0.222	−0.231	−2.349	0.021
θ	6.794	1.698	0.375	4.002	0.000
τ	3.236	10.652	0.031	0.304	0.762
γ	−0.047	0.009	−0.483	−5.454	0.000
δ	−4.469	0.727	−0.527	−6.145	0.000
